# Gut Microbiota Protected Against *pseudomonas aeruginosa* Pneumonia *via* Restoring Treg/Th17 Balance and Metabolism

**DOI:** 10.3389/fcimb.2022.856633

**Published:** 2022-06-16

**Authors:** Long Wen, Lei Shi, Xiang-Long Kong, Ke-Yu Li, Hui Li, Di-Xuan Jiang, Fan Zhang, Zhi-Guo Zhou

**Affiliations:** ^1^ Department of Respiratory and Critical Care Medicine, The First Hospital of Changsha, Changsha, China; ^2^ The Fourth Hospital of Changsha, Changsha, China; ^3^ Department of Anesthesiology, Xiangya Hospital, Central South University, Changsha, China

**Keywords:** gut microbiota, *pseudomonas aeruginosa* pneumonia, Treg/Th17 balance, metabolism, FMT

## Abstract

**Backgrounds and Purpose:**

The theory of “entero-pulmonary axis” proves that pneumonia leads to gut microbiota disturbance and Treg/Th17 immune imbalance. This study is aimed to explore the potential mechanism of fecal microbiota transplantation (FMT) in the treatment of *Pseudomonas aeruginosa* pneumonia, in order to provide new insights into the treatment of pneumonia.

**Methods:**

*Pseudomonas aeruginosa* and C57/BL6 mice were used to construct the acute pneumonia mouse model, and FMT was treated. Histopathological changes in lung and spleen were observed by HE staining. The expression of CD25, Foxp3 and IL-17 was observed by immunofluorescence. The proportion of Treg and Th17 cells was analyzed by flow cytometry. Serum IL-6, LPS, and IFN-γ levels were detected by ELISA. The expression of TNF-α, IFN-γ, IL-6, IL-2, Foxp3, IL-17, IL-10, and TGFβ1 in lung tissue homogenate was detected by qRT-PCR. 16S rRNA sequencing and non-targeted metabolomics were used to analyze gut microbiota and metabolism.

**Results:**

*Pseudomonas aeruginosa* caused the decrease of body weight, food and water intake, lung tissue, and spleen injury in mice with pneumonia. Meanwhile, it caused lung tissue and serum inflammation, and Treg/Th17 cell imbalance in mice with pneumonia. *Pseudomonas aeruginosa* reduced the diversity and number of gut microbiota in pneumonia mice, resulting in metabolic disorders, superpathway of quinolone and alkylquinolone biosynthesis. It also led to the decrease of 2-heptyl-3-hydroxy-4(1H)-quinolone biosynthesis, and the enrichment of Amino sugar and nucleotide sugar metabolism. FMT with or without antibiotic intervention restored gut microbiota abundance and diversity, suppressed inflammation and tissue damage, and promoted an immunological balance of Treg/Th17 cells in mice with pneumonia. In addition, FMT inhibited the aerobactin biosynthesis, 4-hydroxyphenylacetate degradation, superpathway of lipopolysaccharide biosynthesis and L-arabinose degradation IV function of microbiota, and improved amino sugar and nucleotide sugar metabolism.

**Conclusions:**

FMT restored the Treg/Th17 cells’ balance and improved inflammation and lung injury in mice with *Pseudomonas aeruginosa* pneumonia by regulating gut microbiota disturbance and metabolic disorder.

## Introduction

Pneumonia is a heterogeneous and complex disease, which is a major source of illness and death worldwide (Pettigrew et al., 2021). The microbiological diagnosis of pneumonia is challenging and unattainable in a large proportion of cases (Jain et al., 2015). Empiric antibiotics focus on a selection of drugs to cover the main bacterial causes of the disease, whereas the threat of antimicrobial resistance suggests that these empiric and definitive treatments will increasingly be compromised in the coming decades (Metlay et al., 2019). A large number of studies have shown that lung diseases are closely related to gut microbiota. For example, normal upper respiratory tract and gut microbiota prevent pneumonia by preventing the colonization of potential pathogens and regulating the immune response (Zhang et al., 2020; Thibeault et al., 2021). The gut microbiota protects the host during infectious pneumonia by enhancing the phagocytic function of primary alveolar macrophages (Schuijt et al., 2016). Therefore, the gut microbiota regulation of bacterial infectious pneumonia may help to develop new treatment strategies.

Crosstalk between gut microbiota and the lungs, known as the “gut-lung axis,” is critical for immune response and homeostasis in the airways (Tan et al., 2020). Pneumonia may cause an imbalance between helper T (Th17) cells and regulatory T (Treg) cells, which not only plays an important role in the course of pneumonia, but also affects the external organs of the lungs, such as gut metabolism and amino acid metabolites (Liu et al., 2017; Sencio et al., 2020). Prophylactic synbiotics can also prevent ventilator-associated pneumonia in sepsis patients by regulating gut flora and environment (Shimizu et al., 2018). Therefore, exploring the changes in gut metabolism and T cell population ratio in mice with pneumonia can provide strong evidence for the treatment of lung diseases by the gut-lung Axis.

Fecal microbiota transplantation (FMT) can correct the ecological imbalance characterized by chronic C. difficile infection and achieve a seemingly safe, relatively inexpensive, rapid and effective cure in the vast majority of patients treatment (Brandt, 2013). FMT can also affect gut colonization of antibiotic-resistant bacteria in adults, showing potential benefits of non-colonization interventions (Tavoukjian, 2019). FMT can be used to treat inflammatory bowel disease by correcting underlying dysbiosis (Imdad et al., 2018). FMT is one of the main therapeutic strategies for regulating gut microbiota balance, but its role in pneumonia caused by *Pseudomonas aeruginosa* is still unknown (Furman et al., 2021; Yang et al., 2021). The purpose of this study was to study the role of gut microbiota and metabolism in *Pseudomonas aeruginosa* pneumonia, and to explore whether the improvement of gut microbial balance through fecal microbiota transplantation can mediate Treg/Th17 or not and thus improve the symptoms of pneumonia.

## Materials and Methods

### Construction and Grouping of Mouse Pneumonia Model

Twenty 8-week-old adult male C57/BL6 mice were used in this study, which were purchased from Hunan Slyke Jingda Experimental Animal Co., LTD. The whole use of animals was approved by the Medical Ethics Committee of The First Hospital of Changsha and conducted in strict accordance with the national institutes of health guidelines for the care and use of experimental animals (No.2021015). Mice were randomly divided into normal group (Control) and acute pneumonia group (Model), 10 mice/group. Acute pneumonia model in mice was constructed as follows (Felix et al., 2018; Tian et al., 2020): The head of the mouse was fixed on a board, which was tilted at an angle above 50 degrees from the horizontal plane. The front teeth were fixed with silk thread to straighten the neck. The neck hair of mice was removed and the surgical department was disinfected. The surgical scissors cut longitudinally the skin in the middle of the neck, blunt to separate the underlying skin muscles, and expose the muscles surrounding the trachea. Blunt separation of muscles laterally along the muscle longitudinal line exposes the trachea. The syringe was inserted into the trachea 0.5~1 cm towards the heart through the tracheal cartilage annulus, and then injected 75 μL 10^8^ CFU/mL (7.5×10^6^ CFU/mice) of *Pseudomonas aeruginosa* (ATCC 15692) was injected at a slow and uniform speed (van der Windt et al., 2012). Mice were erected and rotated along the longitudinal axis of the body for 3 mins to make the bacterial fluid evenly distributed in both lungs. After being sutured, the mice were placed on an electric blanket to wait for awakening, after which, their body weight, food intake and water intake of mice were measured every day for 7 days. The fecal sample was collected for 16S rRNA gene sequencing, Metabonomics analysis, and FMT. Spleen and lung tissue, as well as the peripheral blood samples were collected for subsequent analysis. Mice were euthanized by CO_2_ inhalation.

### FMT

Forty 8-week-old male C57/BL6 mice were randomly divided into: pneumonia group (Model), pneumonia + FMT group (FMT), pneumonia + antibiotic group (ABX), pneumonia + antibiotic + FMT group (ABX_FMT), 10 mice/group. Broad-spectrum antibiotics [vancomycin (0.5 g/liter), Metronidazole (1 g/liter), and ampicillin (1 g/liter)] in drinking water were used to treat the mice to remove gut microbiota for 21 days, then FMT was transplanted in the ABX group and ABX_FMT group. Thereafter all mice were modeled with or without FMT and observed continuously for 7 days. The specific procedures of FMT were as follows (Schuijt et al., 2016): Faecal pellets from untreated mice were resuspended in PBS (1 faecal pellet/1 mL of PBS). For each experiment, several faecal pellets from different untreated mice were resuspended together in PBS. A total of 200 μL of the resuspended pool faecal material was given by oral gavage to pneumonia modeled mice over 4 consecutive days after antibiotic treatment. After the test, the fecal sample was collected for 16S rRNA gene sequencing and Metabonomics analysis. The body weight, food intake and water intake of mice were measured every day for 7 days. Spleen and lung tissue, as well as the peripheral blood samples were collected for subsequent analysis. The detailed methodology of the experiment was shown in **Supplementary Figure 1**.

### 16S rRNA Gene Pyrosequencing and Data Processing

In this study, Illumina Novaseq PE250 was used for 16S amplicon sequencing to obtain the original data. Qiime (QIIME2-2020.2) and R software (4.0.2) were used to calculate the alpha diversity index (R phyloseq package), visualization of microbial population (VennDiagram package), anosim dimension reduction analysis based on the Bray-Curtis distance (phyloseq/vegan package), and the difference analysis diagram of species abundance based on wald test (R DESeq2 package) (Caporaso et al., 2010; Wang et al., 2012). Microbiata functional enrichment was analyzed by the PICRUSt2 (https://github.com/picrust/picrust2) and MetaCyc databases (https://metacycorg/) (Caspi et al., 2020; Cayetano et al., 2021).

### Metabonomics Analysis

LC-MS/MS analyses were performed using a UHPLC system (1290, Agilent Technologies) with a UPLC BEH Amide column (1.7 μm 2.1×100 mm, Waters) coupled to a TripleTOF 6600 (Q-TOF, AB Sciex) and QTOF 6550 (Agilent). MS raw data files were converted to the mzXML format using ProteoWizard and processed by the R package XCMS (version 3.2). The R package CAMERA was used for peak annotation after XCMS data processing. MetaboAnalyst (Pang et al., 2021) (https://www.metaboanalyst.ca/) and Kyoto Encyclopedia of Genes and Genomes (Kanehisa and Goto, 2000) (KEGG, https://www.kegg.jp/) were used for bioinformatics analysis.

### Hematoxylin-Eosin Staining

Spleen and lung tissues were fixed in 4% paraformaldehyde, and then dehydrated by sucrose solution (20% and 30%). The tissues were dehydrated, embedded in paraffin, sliced successively with a paraffin slicer, connected to the treated slides, and baked at 60°C. Sections were stained with hematoxylin (Wellbio), washed with distilled water, and turned blue with PBS (Wellbio). After that, they were dyed with eosin (Wellbio) and rinsed with distilled water. And then, they were dehydrated with 95~100% gradient alcohol, sealed with neutral gum and observed under a microscope (Motic, BA210T).

### Immunofluorescence

Lung tissue sections were treated for heat repair antigen and washed with 0.01M PBS (pH7.2~7.6) after cooling. The sections were added with 1% periodate acid to inactivate endogenous enzymes. Then the primary antibodies anti-CD25 (bs-0577R, 1:50, Bioss), anti-Foxp3 (22228-1-AP, 1:50, PTG) and anti-IL17 (13082-1-AP, 1:50, PTG) were incubated overnight at 4°C. The sections were dropped with 50-100 μL anti-rabbit-IgG labeled fluorescent antibody (SA00013-2, Proteintech) and incubated at 37°C for 30 min. The sections were visualized with DAB (ZSGB-Bio, China) substrates. The sections were stained, dehydrated, and sealed to be observed by a microscope (Motic, BA210T).

### qRT-PCR

The total RNA was extracted from lung tissues by Trizol (V900483, Sigma). The concentration of total RNA was determined by a UV spectrophotometer. The mRNA Reverse Transcription Kit (CW2569, Kangweishi, Beijing, China) reversely transcribed RNA into cDNA. The expression of target genes was detected by UltraSYBR Mixture (CW2601, Beijing, China) and fluorescence quantitative RCP instrument (PIKOREAL96, Thermo). The 2^-ΔΔCt^ and β-actin were used to calculate the relative expression of TNF-α, IFN-γ, IL-6, IL-2, IL-17, Foxp3, IL-10 and TGFβ1 genes in lung tissue. The gene primer sequences were shown in **Table 1**.

**Table 1:** The primer sequences.

Gene	Sequence	Length
TNF-α	F AGCACAGAAAGCATGATCCG	162bp
	R CACCCCGAAGTTCAGTAGACA	
IFN-γ	F GCCACGGCACAGTCATTGA	201bp
	R TGCTGATGGCCTGATTGTCTT	
IL-6	F GACTTCCATCCAGTTGCCTT	150bp
	R ATGTGTAATTAAGCCTCCGACT	
IL-2	F GTGCTCCTTGTCAACAGCG	171bp
	R GGGGAGTTTCAGGTTCCTGTA	
FOXP3	F CTCCAATCCCTGCCCTTGACC	130bp
	R ACATCATCGCCCGGTTTCCA	
IL-17	F AGACTACCTCAACCGTTCCAC	229bp
	R CACCAGCATCTTCTCGACCC	
IL-10	F GTTCCCCTACTGTCATCCCC	149bp
	R AGGCAGACAAACAATACACCA	
TGFβ1	F CTCCCGTGGCTTCTAGTGC	133bp
	R GCCTTAGTTTGGACAGGATCTG	
β-actin	F ACATCCGTAAAGACCTCTATGCC	223bp
	R TACTCCTGCTTGCTGATCCAC	

### Flow Cytometry

Spleen tissue was collected and digested by enzymes to obtain the mononuclear cells suspension. Then the cells were purified by a monocyte isolation kit (P5390, Solarbio) and differential adherent method was utilized to obtain splenic monocytes. 1×10^6^/100 μL cells were taken into a 1.5 mL EP tube, supplemented with culture medium and 1 μL Cell Stimulation Cocktail (Plus protein Transport inhibitors) 500× (00-4975-93, EBiosciences) in a 37°C incubator for 4h. Cells were washed with 1mL PBS, centrifuged at 350g for 5 min, and the supernatant was discarded. Fixation/Permeabilization (00-5123-43, eBiosciences) was concentrated by 1:3 in Fixation/perm Diluent dilution into 1× working liquid. Then resuspended cells at room temperature away from light to fix broken film for 30 min. The 10× Permeabilization Buffer was diluted with deionized water at 1:9 to 1× working solution to resuspended cells. The resulting resuspension was centrifuged at 350g for 5 min, and the supernatant was discarded. The cell precipitates were suspended with 100 μL PBS. Th17 cells were assays, respectively, with 0.125μg CD4 (11-0043-82, eBiosciences) and 0.125μg IL-17A (17-7177-81, eBiosciences), respectively. Treg cells were assays, respectively, with 0.125μg CD4 (11-0043-82, eBiosciences), 0.125μg CD25 (25-0251-82, eBiosciences) and 0.25μg Foxp3 (35-5773-82, eBiosciences), respectively. Subsequently, mixed them well and incubated for 30 min at room temperature away from light. The cells were resuscitated by PBS and detected by flow cytometry (A00-1-1102, Beckman).

### ELISA

Peripheral blood samples of mice in different treatment groups were collected. Then the samples were centrifuged at 2-8°C 1000g to collect serum. Serum IFN-γ (CSB-E04578m, CUSABIO), IL-6 (CSB-E04639m, CUSABIO) and LPS (CSB-E13066m, CSB-E13066m, CUSABIO) were detected by the multifunctional analyzer (MB-530, HEALES) according to the manufacturer’s instructions. All samples were tested 3 times.

### Data Statistics and Analysis

Graph Prism (version 8.0) and R software (version 3.1.0) were used for statistical analysis. Statistical significance between two groups within experiments was determined by unpaired two-tailed Student’s t-tests and among more than two groups using ANOVA. P<0.05 was considered statistically significant.

## Results

### Gut Microbiota Disorder and Functional Change Occurred in Mice With Acute Pneumonia

**Figure 1:**
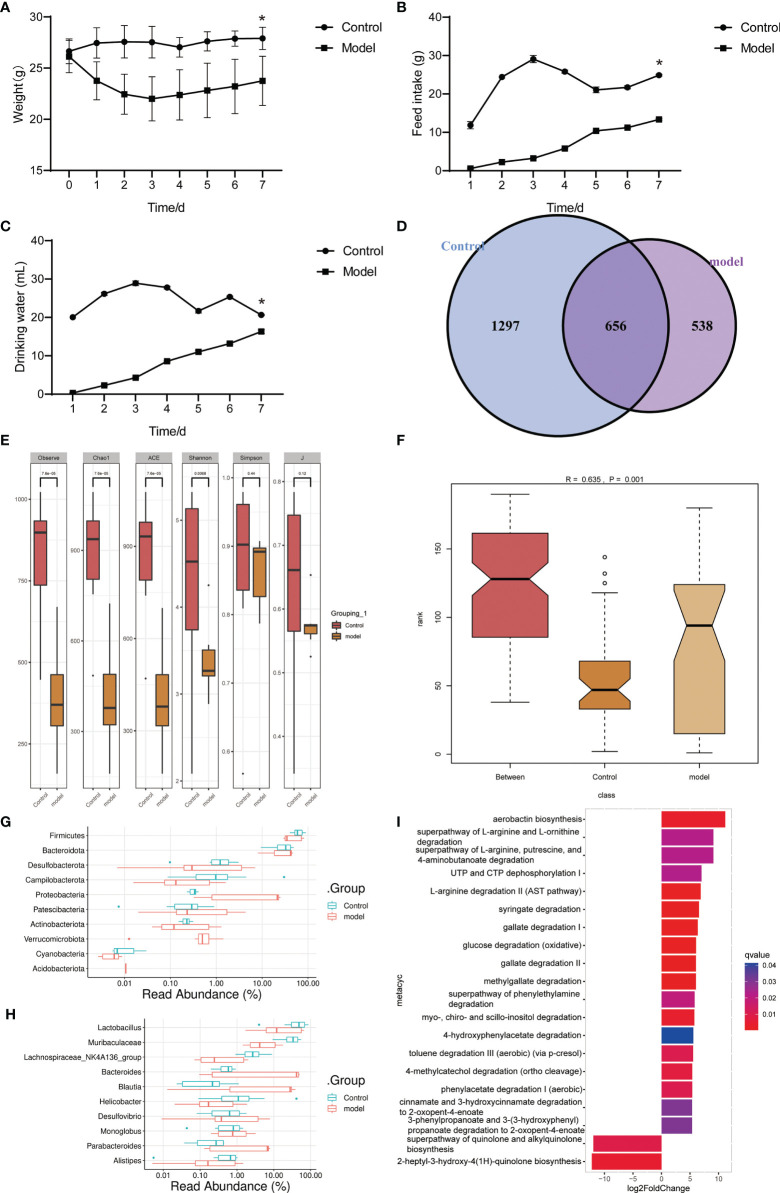
Gut microbiota and functional changed in mice with acute pneumonia (n=10 mice/group). **(A–C)** Body weight, food and water intake of mice were observed and monitored for 7 days. **(D)** Venn diagram showed changes in microbial population. **(E)** Alpha diversity index. **(F)** Beta diversity index. **(G, H)** The histogram showed microbiota abundance at the phylum and genus level. **(I)** The annotation of microbiota functional pathway. N=5 sample/group for 16S rRNA sequencing. ^*^ *P* < 0.05 vs Control group.

We used *Pseudomonas aeruginosa* to construct a mouse model of acute pneumonia finding that the weight of mice decreased, and the intake of food and water decreased significantly in the model group (**Figures 1A–C**). Gut microbiota sequencing analysis showed that the number and alpha diversity of gut microbiota were decreased in the model group (**Figures 1D, E**). The beta diversity index indicated significant differences between groups (**Figure 1F**). At the phylum level, the abundance of Firmicutes, Desulfobacterota, Campilobacterota, Actinobacteriota and Cyanobacteria were decreased, and the abundance of Proteobacteria, Verrucomicrobiota and Acidobacteriota were increased in the gut of mice in the model group (**Figure 1G**). Interestingly, at the genus level, the abundance of *Lactobacillus*, *Muribaculaceae*, *Lachnospiraceae_NK4A136_group*, *Helicobacter* and *Alistipes* were decreased, and the abundance of *Bacteroides*, *Blautia*, and *Parabacteroides* were increased in the gut of mice in the model group (**Figure 1H**). Microbial functional analysis revealed the superpathway of quinolone and alkylquinolone biosynthesis and 2-heptyl-3-hydroxy-4(1H)-quinolone biosynthesis pathways were significantly decreased in the gut microbiota of mice in model group (**Figure 1I**). These results suggested that gut microbiota in mice with pneumonia is disturbed, accompanied by a decrease in microbial diversity and number, and function changes.

### Metabolites and Functional Changes in Mice With Acute Pneumonia

**Figure 2:**
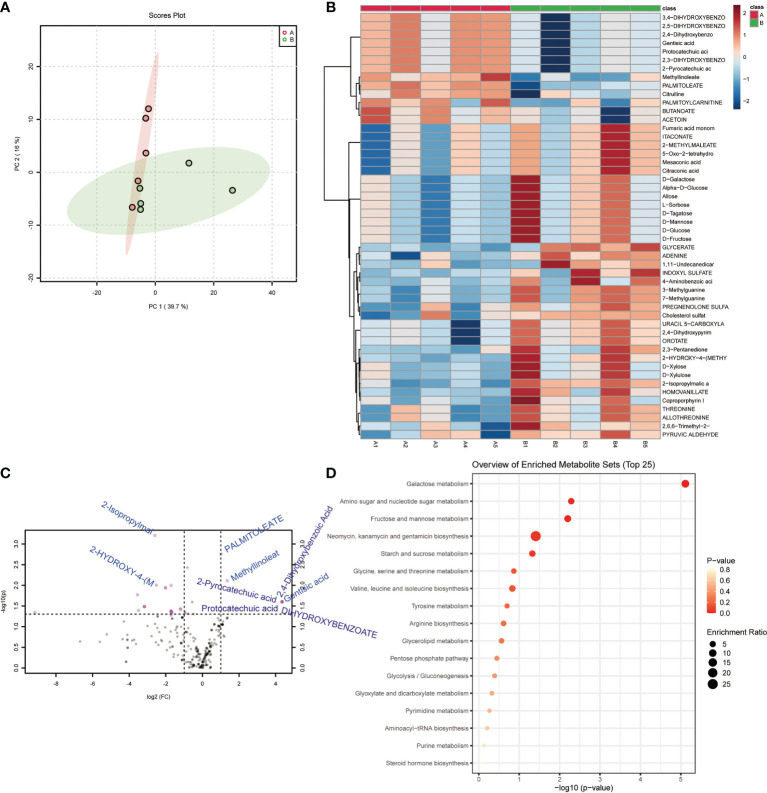
Gut metabolites and functional changed in mice with acute pneumonia (n=10 mice/group). **(A)** PCA analysis of sample differences between control group and model group. **(B)** Heatmap showed changes in metabolite abundance. **(C)** Volcanograms showed differential metabolites. **(D)** Bubble map showed metabolite functional pathways. N=5 sample/group for Non-targeted Metabolomics Sequencing.

Principal Component Analysis suggested that the samples in the control and model groups were significantly distinguished (**Figure 2A**). Comparison analysis of metabolite found that 2-Isopropylmalic acid, 2-HYDROXY-4-(METHYLTHIO)BUTANOATE, ADENINE, 3-Methylguanine, 7-Methylguanine, HOMOVANILLATE, 2,4-Dihydroxypyrimidine-5-carboxylic acid, OROTATE, URACIL 5-CARBOXYLATE, THREONINE, ALLOTHREONINE, 2,3-Pentanedione, Alpha-D-Glucose, Allose, D-Galactose, D-Tagatose, D-Glucose, D-Fructose, D-Mannose, INDOXYL SULFATE and L-Sorbose were significantly increased in model group (P<0.05) (**Figures 2B, C**). PALMITOLEATE, Methyllinoleate, Gentisic acid, 2,3-DIHYDROXYBENZOATE, 2-Pyrocatechuic acid, Protocatechuic acid, 2,5-DIHYDROXYBENZOATE, 2,4-Dihydroxybenzoic Acid, 3,4-DIHYDROXYBENZOATE and Citrulline were significantly decreased in model group (P<0.05) (**Figures 2B, C**). Functional annotation showed that Galactose metabolism, Fructose and mannose metabolism and Amino sugar and nucleotide sugar metabolism were significantly enriched (**Figure 2D**). In particular, C00984 (D-galactose), C00267 (alpha-D-glucose) and C02336 (D-Fructose) were significantly enriched in Amino sugar and nucleotide sugar metabolism (**Figures 2B, D** **)**. These results suggested that the gut metabolite profile and function of acute pneumonia mice were altered and involved in the course of the disease.

### Acute Pneumonic Mice Induced Systemic Inflammation and Treg/Th17 Cell Imbalance

**Figure 3:**
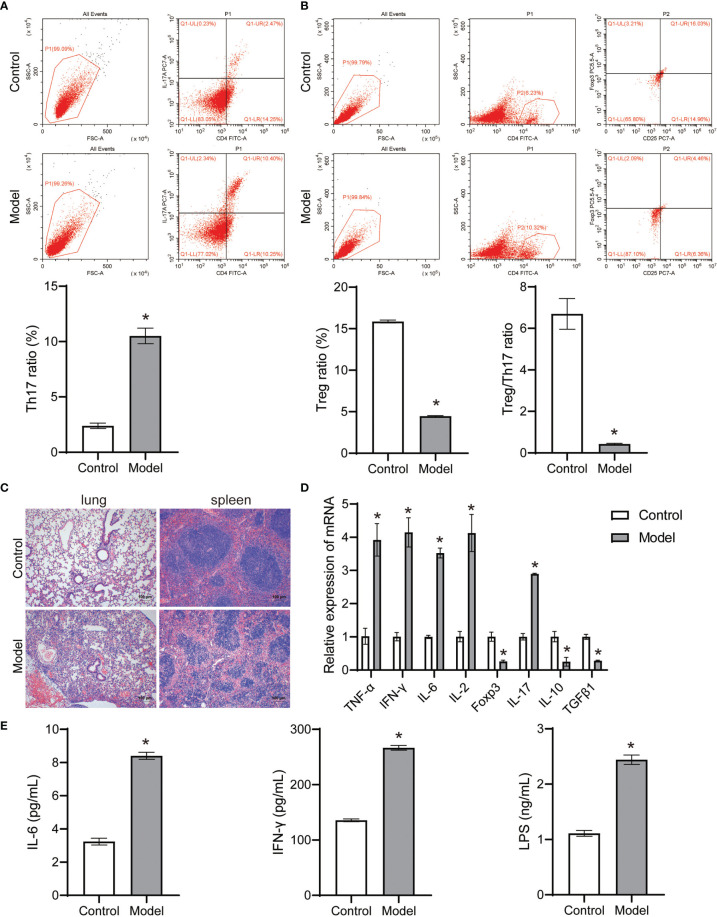
*Pseudomonas aeruginosa* induced immune differentiation and inflammation of mice (n=10 mice/group). **(A, B)** The Treg and Th17 cells ratio in spleen tissues were analyzed by flow cytometry. **(C)** Histopathological changes of lung and spleen were observed by HE staining (100×). **(D)** The expression of TNF-α, IFN-γ, IL-6, IL-2, Foxp3, IL-17, IL-10 and TGFβ1 in lung tissue homogenate was detected by qRT-PCR. **(E)** Serum IL-6, LPS and IFN-γ levels were detected by ELISA. ^*^ *P* < 0.05 vs control group. Each sample was replicated 3 times.

The theory of “Lung homing T-cell” proved that continuous inhalation of antigen for 7 days in normal mice resulted in continuous presentation of lymph nodes, but not lungs (Wikstrom et al., 2010). Environmental stress induces a substantial re-distribution of T-cells within lymphoid and non-lymphoid organs (Kruger and Mooren, 2007). A uniform response pattern seems to exist with a decrease in lymphocyte numbers in the spleen which is accompanied by an increase in lymphocytes in lung, bone marrow and Peyer’s patches (Kruger and Mooren, 2007). The onset of acute pneumonia induced immune differentiation of CD4+T cells in the spleen of mice, accompanied by an increase in Th17 cell ratio and a decrease in Treg cell ratio, as well as a severe decrease in Treg/Th17 cell ratio (**Figures 3A, B**). Pathological observation showed that the lung tissue and spleen tissue of the control group were intact, and the nucleus was clearly visible, and the contrast between the nucleus and cytoplasm was distinct (**Figure 3C**). The lung tissue and spleen tissue of acute pneumonia mice were obviously damaged, and the nucleus disappeared, and the cytoplasm remained red (**Figure 3C**). Acute pneumonia increased the expression of TNF-α, IFN-γ, IL-6, IL-2 and IL-17 genes, and decreased the expression of Foxp3, IL-10, and TGFβ1 genes in lung tissue homogenate of mice (**Figure 3D**). Acute pneumonia also increased the levels of IL-6, LPS and IFN-γ in the serum of mice (**Figure 3E**). These studies demonstrated that lung and spleen tissue damage, increased systemic inflammation and dynamic imbalance of Treg/Th17 cells occurred in mice with acute pneumonia.

### FMT Improved Gut Microbial Diversity and Function in Mice With Acute Pneumonia

**Figure 4:**
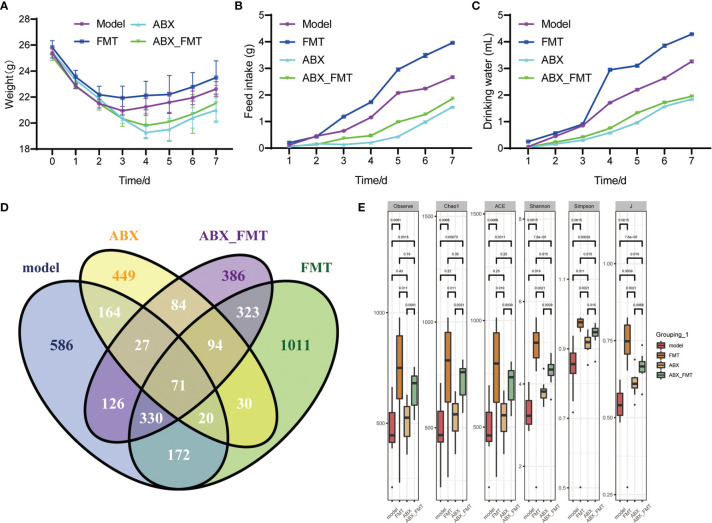
FMT improved physiological characterization and gut microbiota diversity in mice with acute pneumonia (n=10 mice/group). **(A-C)** Body weight, food and water intake of mice were observed and monitored for 7 days. **(D)** The quantitative analysis of common and endemic microbiota. **(E)** Alpha diversity index. N=5 sample/group for 16S rRNA sequencing.

**Figure 5:**
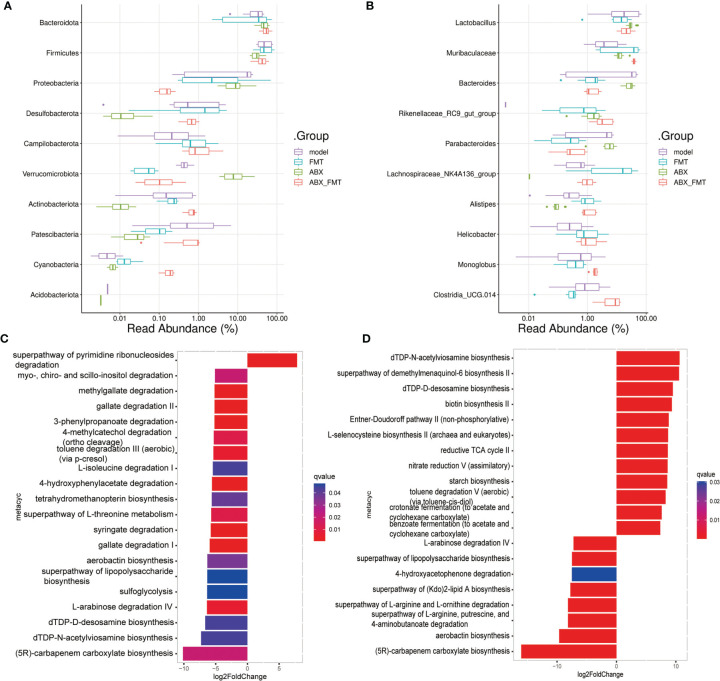
FMT improved the function of gut microbiota in mice with acute pneumonia. **(A, B)** The histogram showed microbiota abundance at the phylum and genus level. **(C)** Differences in gut microbial functional pathways between model group and FMT group. **(D)** Differences in gut microbial functional pathways between ABX group and ABX_FMT group. N=5 sample/group for 16S rRNA sequencing.

To investigate the role of gut microorganisms in mice with acute pneumonia, FMT was treated with or without antibiotic intervention. We found that FMT intervention evidently improved the weight, food intake and drinking water of mice with acute pneumonia (**Figures 4A–C**). ABX with or without FMT intervention reduced weight, food intake, and drinking water of mice with acute pneumonia (**Figures 4A–C**). Furthermore, ABX intervention significantly reduced the gut microbial number of mice with acute pneumonia (**Figure 4D**). FMT with or without antibiotic intervention significantly increased the number and diversity of gut microorganisms in mice with acute pneumonia (**Figures 4D, E**). At the phylum level, FMT has significantly reduced the abundance of Proteobacteria, Verrucomicrobiota and Acidobacteriota, and increased the abundance of Firmicutes, Desulfobacterota, Campilobacterota, Cyanobacteria and Actinobacteriota (**Figure 5A**). At the genus level, FMT significantly reduced the abundance of *Lactobacillus*, *Bacteroides* and *Parabacteroides*, and increased the abundance of *Muribaculaceae*, *Lachnospiraceae_NK4A136_group*, *Alistipes* and *Helicobacter* (**Figure 5B**). In addition, FMT with or without antibiotic intervention significantly inhibited gut microbiota’s aerobactin biosynthesis, 4-hydroxyphenylacetate degradation, superpathway of lipopolysaccharide biosynthesis and L-arabinose degradation IV pathways in mice with acute pneumonia (**Figures 5C, D**). These results suggested that FMT could improve the functional pathways related to gut microbial diversity in mice with acute pneumonia, which may be correlated with the diversity of aerobic or anaerobic microorganisms and LPS biosynthesis in mice with acute pneumonia.

### FMT Improved Metabolism and Function in Mice With Acute Pneumonia

**Figure 6:**
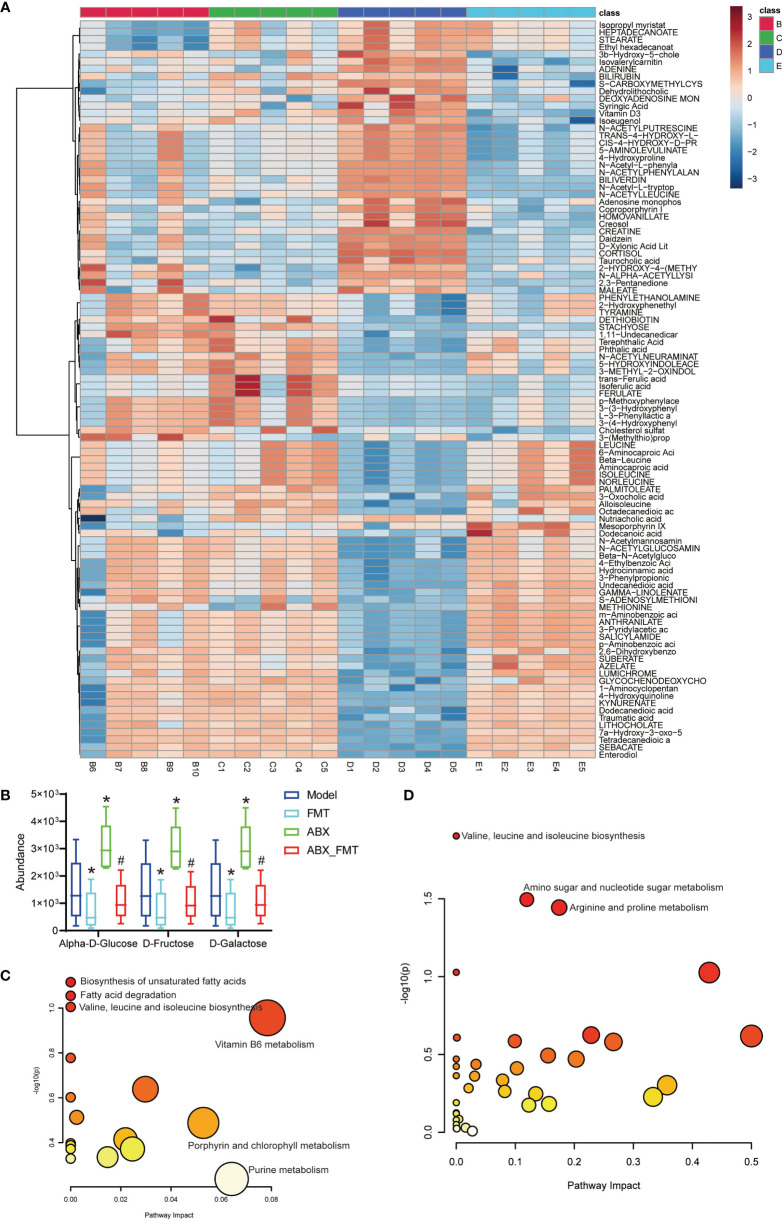
FMT improved gut metabolism and function in mice with acute pneumonia (n=10 mice/group). **(A)** Heatmap showed changes of metabolites in mice with acute pneumonia. **(B)** The abundance of D-galactose, alpha-D-glucose and D-Fructose metabolites. **(C)** KEGG annotation was used to analyze the metabolite functions of model group and FMT group. **(D)** KEGG annotation was used to analyze the metabolite functions of ABX group and ABX_FMT group. *P < 0.05 vs control group; #P < 0.05 vs ABX group. N=5 sample/group for Non-targeted Metabolomics Sequencing.

FMT improved gut metabolic structure and significantly reduced D-galactose, Alpha-D-glucose and D-fructose abundance in mice with or without antibiotic intervention (**Figures 6A, B**). KEGG functional enrichment analysis indicated that FMT inhibited the biosynthesis of unsaturated fatty acids, fatty acid degradation and valine, leucine and isoleucine biosynthesis pathways in mice with acute pneumonia (**Figure 6C**). In the presence of antibiotics, FMT significantly inhibited Valine, Leucine and isoleucine biosynthesis (P =0.012), Amino sugar and nucleotide sugar metabolism (P =0.032) and Arginine and proline metabolism (P =0.036) in mice with acute pneumonia (**Figure 6D**). These results suggested that FMT was able to improve metabolism and function in mice with acute pneumonia.

### FMT Inhibited Inflammation and Restored Treg/Th17 Cell Balance in Mice With Acute Pneumonia

**Figure 7:**
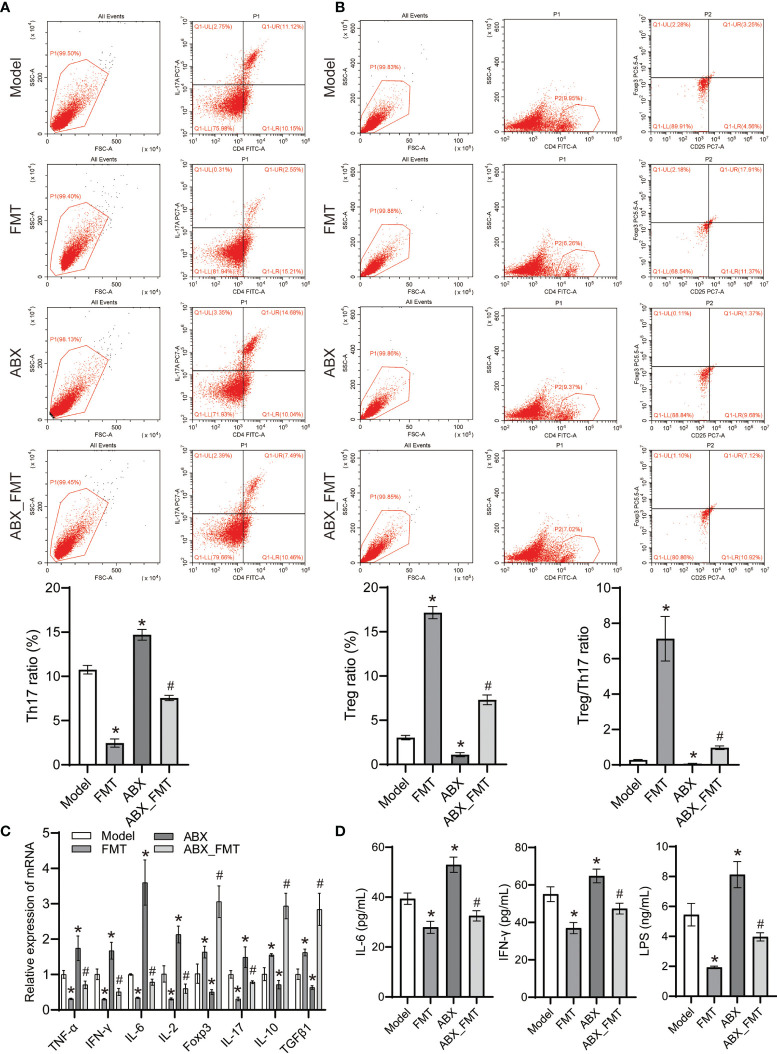
FMT affected Treg cell differentiation and inhibits inflammation in mice with acute pneumonia (n=10 mice/group). **(A-B)** The Treg and Th17 cells ratio in spleen tissues were detected by flow cytometry. **(C)** The expression of TNF-α, IFN-γ, IL-6, IL-2, Foxp3, IL-17, IL-10 and TGFβ1 in lung tissue homogenate was detected by qRT-PCR. **(D)** Serum IL-6, LPS and IFN-γ levels were detected by ELISA. *P<0.05 vs control group; #P < 0.05 vs ABX group. Each sample was replicated 3 times.

FMT reduced the proportion of Th17 cells but increased the proportion of Treg cells and Treg/Th17 in spleen tissue of mice with and without antibiotic intervention (**Figures 7A, B**). FMT reduced the expression of TNF-α, IFN-γ, IL-6, IL-2 and IL-17 genes, increased the expression of Foxp3, IL-10 and TGFβ1 genes in lung tissue homogenate (**Figure 7C**). FMT has also shown decreased the levels of IL-6, LPS and IFN-γ in the serum of mice with acute pneumonia (**Figure 7D**). These results suggested that FMT improved inflammation and Treg/Th17 cell imbalance in mice with acute pneumonia.

### FMT Promoted Immunity and Improved Lung Injury in Mice With Acute Pneumonia

**Figure 8:**
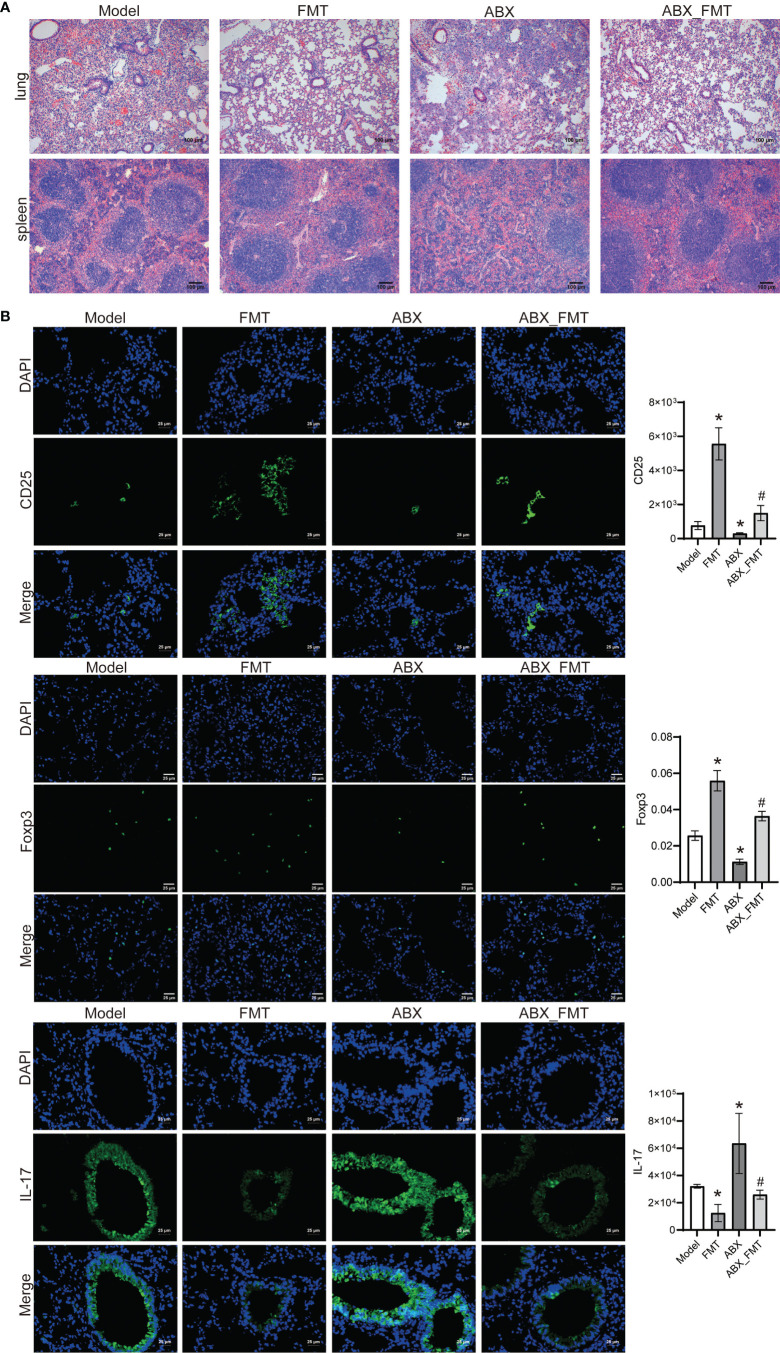
FMT improved tissue injury and immunity in mice with acute pneumonia (n=10 mice/group). **(A)** HE staining was used to observe the pathological injury of lung and spleen tissues (100×). **(B)** The expression of CD25, Foxp3 and IL-17 in lung tissues were detected by Immunofluorescence (400×). *P < 0.05 vs control group; #P < 0.05 vs ABX group. Each sample was replicated 3 times.

With or without antibiotic intervention, the lung and spleen tissues of acute pneumonia mice in the FMT group were intact, the nucleus was clearly visible. At the same time, contrast between nucleus and cytoplasm was distinct (**Figure 8A**). Immunofluorescence staining showed that FMT increased the CD25 and Foxp3 expressions, and decreased the IL-17 expression in mice with and without antibiotic intervention (**Figure 8B**). These results demonstrated that FMT promoted Treg cell immunity and improved lung injury in mice with acute pneumonia.

## Discussion

Pneumonia is considered to be one of the leading causes of death worldwide and its outcome depends on appropriate antibiotic treatment and the effectiveness of the host immune response (Schirm et al., 2016). *Pseudomonas aeruginosa* induces an increase in the inflammatory cytokines IL-17, IL-6, and TNF-α in the lung tissue of mice, resulting in an increase in Th17 cells (Lou et al., 2021). Th17 cells protect mucosal surfaces, including the lungs, from Gram-negative pathogens (Wu et al., 2012; Schaefers et al., 2018). Our study found that mice infected with pneumococcal infection had decreased body weight, as well as food and water intake, lung tissue and spleen are damaged. Their lung tissue and serum inflammation increase; Treg/Th17 cells become imbalanced; gut microbial number and diversity decrease, and their gut metabolic disorders, too. Stimulation of *Pseudomonas aeruginosa* or its components triggers IL-17 responses in thymocytes and spleen cells (Weber et al., 2016). These studies have proved that *pseudomonas aeruginosa* infection leads to systemic inflammation and Th17 cell response in mice, and exogenous intervention of Th17 cell immunity may be an effective method to alleviate pneumonia.

Hospital-acquired and community-acquired bacterial infections have placed a heavy burden on global health systems (Zhang et al., 2019). Our study found that *pseudomonas aeruginosa* infection resulted in inhibition of the superpathway of quinolone and alkylquinolone biosynthesis and 2-heptyl-3-hydroxy-4(1H)-quinolone biosynthesis of gut microorganisms in mice, proving that *pseudomonas aeruginosa* infection resulted in weakened anti-infection or anti-inflammatory ability of gut symbiotic bacteria in mice. *Pseudomonas aeruginosa* has a typical quorum sensing system, including, but not limited to, the top LasI-LasR pathway and the downstream RhlI-RhlR, PqsABCDE-PqsR and AmbBCDE-IqsR pathways (Whiteley et al., 2017). PqsABCDE can synthesize 2-heptyl-4-quinolone using anthranilic acid as precursor, and synthesize 2-heptyl-3-hydroxy 4-quinolone through PqsH processing in the PqsABCDE-PqsR pathway (Mcglacken et al., 2010; Lee and Zhang, 2015). *Pseudomonas aeruginosa* produces 2-heptyl-3-hydroxy-4 (1H) -quinolone, a quorum sensing signal that regulates many virulence genes, comprising those involved in iron clearance (Diggle et al., 2007). These findings suggested that quorum sensing induced functional changes of gut microorganisms in mice by *Pseudomonas aeruginosa* invasion may be a potential intervention target for the treatment of *Pseudomonas aeruginosa* infection pneumonia.

Gut symbiotic segmental filamentous bacteria can provide much-needed protection in immunocompromised hosts, in part against bacterial pneumonia by promoting neutrophils regression after lung infection (Felix et al., 2018). Gut microbiota from alcohol-fed mice significantly impaired clearance of pathogenic bacteria, demonstrating that alcohol feeding and alcohol-related gut microbiota disorders impair lung host defenses against pathogenic bacteria (Samuelson et al., 2019). Probiotics reduce proinflammatory cytokine gene expression in the colon and spleen after pneumonia and prevent downstream organ dysfunction due to systemic inflammation (Khailova et al., 2017). Changes in microbiota can alter susceptibility to pulmonary infection by pathogenic bacteria independently of host genotypes (Dabrowski et al., 2019). Gut microbiome dysregulation impairs hosts’ defenses against *pseudomonas aeruginosa* pneumonia, presenting as an increase of bacterial burden and transmission, which may be associated with defective γδ T17 cells and downstream neutrophilic responses (Wang et al., 2020). Our study showed that FMT with or without antibiotic intervention restored gut microbiome diversity, suppressed inflammation and tissue damage, and promoted the immunological balance of Treg/Th17 cells in mice with pneumonia. These findings suggested that the transplantation of hosts’ gut symbiotic bacteria could improve the basic characterization of the disease and may be a new strategy for the treatment of infectious pneumonia.

1, 5-dehydrate-d-fructose (1, 5-AF) is a monosaccharide formed from starch and glycogen that has antioxidant and antibacterial activities and inhibits cytokine release by attenuating NF-κb activation in LPS-stimulated mice (Meng et al., 2009b). Pretreatment with 1, 5-AF inhibited the protein and mRNA expression of iNOS in lung tissues, and upregulated the IL-10 level in serum of mice with acute pneumonia. Meanwhile, the inhibition effect seemed to be prolonged and enhanced by the production of IL-10 (Meng et al., 2009a). It is known that 1, 5-AF was efficiently prepared from D-fructose *via* a region-specific 1, 5-dehydrated ring formation and subsequent deprotection of 2, 3-O-isopropyl-1-o-methyl (methylphenyl) sulfonyl-D-pyrantose (Dekany et al., 2007). Exogenous supplementation of 1, 5-AF altered the composition and metabonomics of gut microbiota, increased the proportion of *Faecalis prausnitzii*, and enriched genes related to the biosynthesis of nicotinamide adenine dinucleotide (Ito et al., 2021). These studies have proved that gut microbiota may synthesize 1, 5-AF by consuming D-fructose, and then play antibacterial and immunomodulatory roles. Our results found that FMT significantly depleted D-galactose, Alpha-D-glucose and D-fructose in the gut of mice with or without antibiotic intervention. We hypothesized that the metabolic transformation of D-fructose may take place in the gut tract of the host and participate in the functional regulation of host metabolism and inflammation. However, the specific mechanism still needs to be further exploring.

Toxins, LPS and proteases secreted by *pseudomonas aeruginosa* are key factors causing acute *pseudomonas aeruginosa* pneumonia (Riquelme and Prince, 2020). The cytokine pattern induced by *Pseudomonas aeruginosa* infection in the thymus and spleen is mainly mediated by LPS of the outer membrane, but other components of the bacterium also influence cytokine secretion (Weber et al., 2016). LPS treatment can polarize naive CD4+ T cells into Th17 and Treg cells and affect Th17/Treg balance and may predispose to Th17 responses (Yuan et al., 2020). Pulmonary Th17/Treg imbalance is closely related to lung inflammation and lung injury (Chen et al., 2020). Our study found that FMT inhibited the aerobactin biosynthesis, 4-hydroxyphenylacetate degradation, superpathway of lipopolysaccharide biosynthesis and L-arabinose degradation IV functions of gut microbiota in mice with *Pseudomonas aeruginosa* pneumonia, proving that FMT can treat pneumonia by improving gut microenvironment and repairing Treg/Th17 balance.

## Data Availability Statement

The datasets presented in this study can be found in online repositories. The names of the repository/repositories and accession number(s) can be found as follows: https://www.ncbi.nlm.nih.gov/sra/PRJNA808805.

## Ethics Statement

This animal study was reviewed and approved by the Ethics Committee of Changsha First Hospital.

## Author Contributions

LW, LS, X-LK, K-YL, HL, D-XJ, FZ and Z-GZ designed the study, performed the research, analyzed data, and wrote the paper. LW, LS and X-LK contributed equally to this paper. They are co-first authors. All authors contributed to the article and approved the submitted version.

## Funding

This work was supported by the key research and development projects of the department of science and technology of Hunan Province (No.2022SK2047), Natural Science Foundation of Hunan Province (No. 2018JJ2452), and the Natural Science Foundation of Hunan Province (No.2019JJ50969).

## Conflict of Interest

The authors declare that the research was conducted in the absence of any commercial or financial relationships that could be construed as a potential conflict of interest.

## Publisher’s Note

All claims expressed in this article are solely those of the authors and do not necessarily represent those of their affiliated organizations, or those of the publisher, the editors and the reviewers. Any product that may be evaluated in this article, or claim that may be made by its manufacturer, is not guaranteed or endorsed by the publisher.

## Supplementary Material

Supplementary Figure 1 The methodology of the experiment.
