# Supplementary figures and images for: Gut Microbiota Protected Against pseudomonas aeruginosa Pneumonia via Restoring Treg/Th17 Balance and Metabolism
Source: Front Cell Infect Microbiol. 2022 Jun 16;12:856633. doi: 10.3389/fcimb.2022.856633 (PMC9243233; doi:10.3389/fcimb.2022.856633)

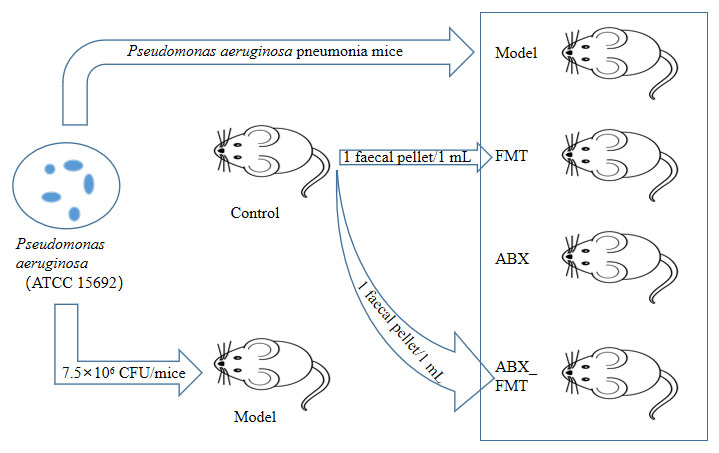

Supplement: Supplementary Figure 1 — The methodology of the experiment. [file Image_1.jpg]
